# Emergence of prions selectively resistant to combination drug therapy

**DOI:** 10.1371/journal.ppat.1008581

**Published:** 2020-05-18

**Authors:** Cassandra M. Burke, Kenneth M. K. Mark, Judit Kun, Kathryn S. Beauchemin, Surachai Supattapone

**Affiliations:** 1 Department of Biochemistry and Cell Biology, Geisel School of Medicine at Dartmouth, Hanover, New Hampshire, United States of America; 2 Department of Medicine, Geisel School of Medicine at Dartmouth, Hanover, New Hampshire, United States of America; Creighton University, UNITED STATES

## Abstract

Prions are unorthodox infectious agents that replicate by templating misfolded conformations of a host-encoded glycoprotein, collectively termed PrP^Sc^. Prion diseases are invariably fatal and currently incurable, but oral drugs that can prolong incubation times in prion-infected mice have been developed. Here, we tested the efficacy of combination therapy with two such drugs, IND24 and Anle138b, in scrapie-infected mice. The results indicate that combination therapy was no more effective than either IND24 or Anle138b monotherapy in prolonging scrapie incubation times. Moreover, combination therapy induced the formation of a new prion strain that is specifically resistant to the combination regimen but susceptible to Anle138b. To our knowledge, this is the first report of a pathogen with specific resistance to combination therapy despite being susceptible to monotherapy. Our findings also suggest that combination therapy may be a less effective strategy for treating prions than conventional pathogens.

## Author summary

Prions are unusual infectious agents that lack DNA or RNA blueprints and cause fatal brain diseases that are currently incurable. Combining two or more drugs has proven to be an effective strategy for treating other infectious agents and cancer cells, and here we evaluated the effectiveness of treating experimental prion disease with a combination of two oral drugs previously shown to prolong the lives of prion-infected mice. Our results show that the combination regimen was no more effective than either of the individual drugs. Combination therapy induced the formation of a new prion strain that is specifically resistant to the combination regimen (i.e. resistant to the combination but not to the individual drug give alone). To our knowledge, this is the first report of an infectious agent with selective resistance to combination therapy in the absence of resistance to an individual component, and suggests that combination therapy may not be a good strategy for combating prions because of their unique malleability.

## Introduction

Prion diseases, such as Creutzfeldt-Jakob disease (CJD) in humans, Chronic Wasting Disease (CWD) in cervids, and bovine spongiform encephalopathy (BSE) in cattle, are a group of invariably fatal neurodegenerative diseases that are caused by unorthodox infectious agents that contain misfolded conformers (collectively termed PrP^Sc^) of a host glycoprotein (PrP^C^). Although prions lack replicating nucleic acid genomes, they can faithfully propagate as distinct self-replicating strains with specific clinical, neuropathological, and biochemical characteristics[1]. Unfortunately, there are currently no clinically useful treatments available for prion disease, and therefore it is imperative to develop and evaluate new therapeutic strategies[2–4].

There are three major barriers to treating prion disease in clinical settings. First, patients are typically not diagnosed until a late stage of disease progression, when neurological symptoms have begun. However, all anti-prion therapies are essentially ineffective at this late stage due to the large burden of accumulated PrP^Sc^[3]. Second, prions are composed of an assortment of conformational PrP^Sc^ variants termed “quasi-species”[5–7]. When prion-infected animals are treated with compounds that target PrP^Sc^ replication, drug-resistant PrP^Sc^ quasi-species emerge, resulting in treatment failure[8, 9]. Indeed, these two problems (large PrP^Sc^ burden and the emergence of drug-resistant PrP^Sc^ quasi-species) are closely related, since having a high rate of PrP^Sc^ formation increases the probability of generating a drug-resistant PrP^Sc^ conformer. Finally, some drugs that are effective at treating one prion strain may be ineffective at treating or even facilitate the propagation of a different strain[10, 11]

Historically, these same problems have also hindered treatment of other infectious diseases and cancer. Specifically, initial attempts using single drugs to treat patients with a large burden or drug-resistant forms of bacteria, viruses, or tumor cells often failed[12, 13]. Fortunately, it was discovered that combination chemotherapy is much more effective in these settings; some specific examples include the discovery of successful multi-drug regimens for tuberculosis[14] and Hodgkin lymphoma[15]. Synergistic combination regimens containing drugs with different molecular targets select for pathogens or cancer cells that harbor multiple protective mutations. Such multi-drug-resistant mutants occur relatively infrequently in treatment-naïve patients; and even when they do occur, the accumulation of multiple mutations often compromises the pathogen’s fitness, providing a clinical benefit.

Here, we evaluate the effect of combination chemotherapy in a mouse model of prion disease. For our study, we chose two structurally distinct chemical compounds, IND24 and Anle138b, both which are known to be orally bioavailable and effective in extending incubation times in mice infected with RML prions[8, 16].

## Results

### Combination therapy does not prolong incubation time relative to monotherapy

**Fig 1:**
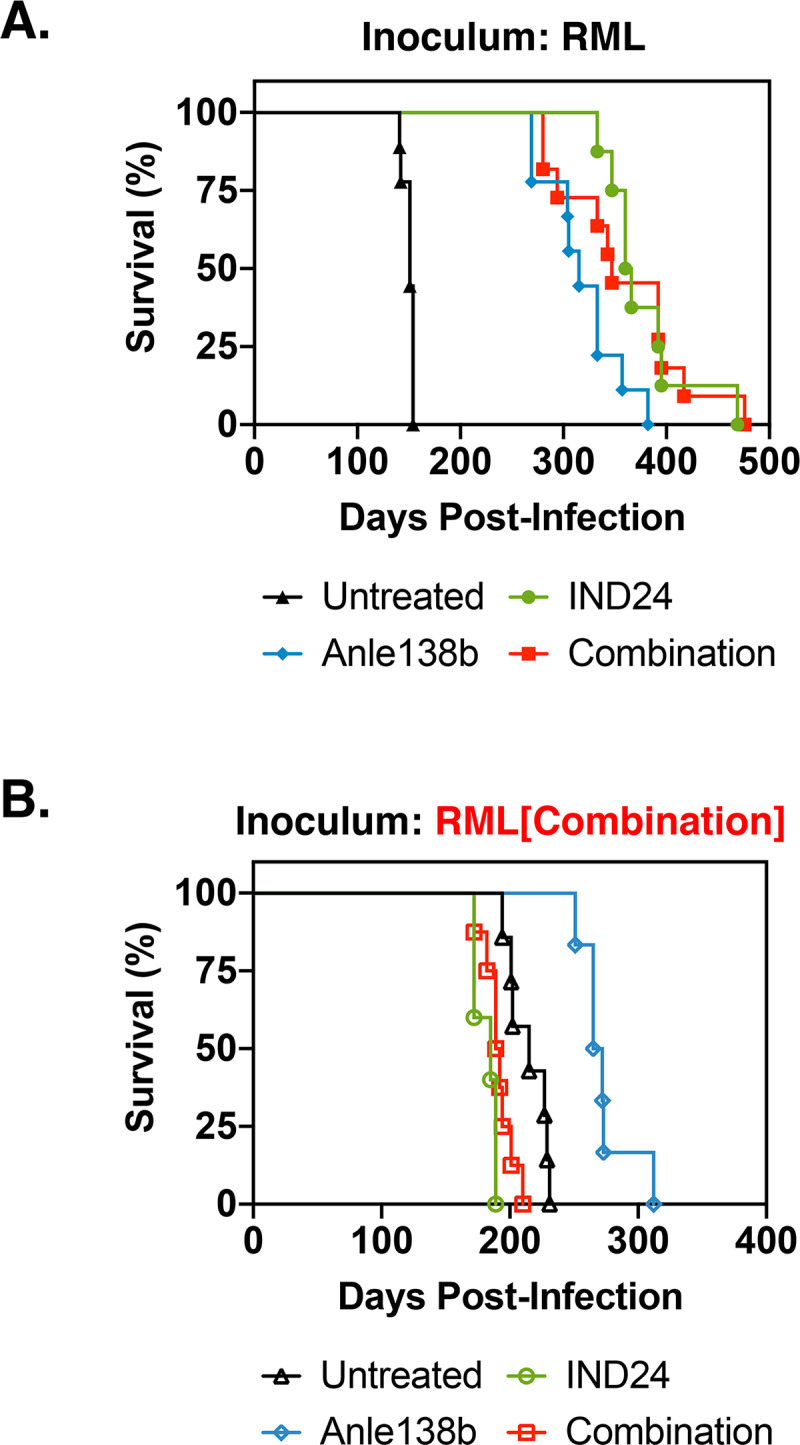
RML inoculations of control- or experimentally- treated mice. Kaplan*-*Meier survival plots of each treatment group in mice inoculated with either **(A)** drug-naïve RML or **(B)** combination chemotherapy-resistant prions (i.e. serial passage of prions from mice originally inoculated with RML and treated with combination therapy).

**Fig 2:**
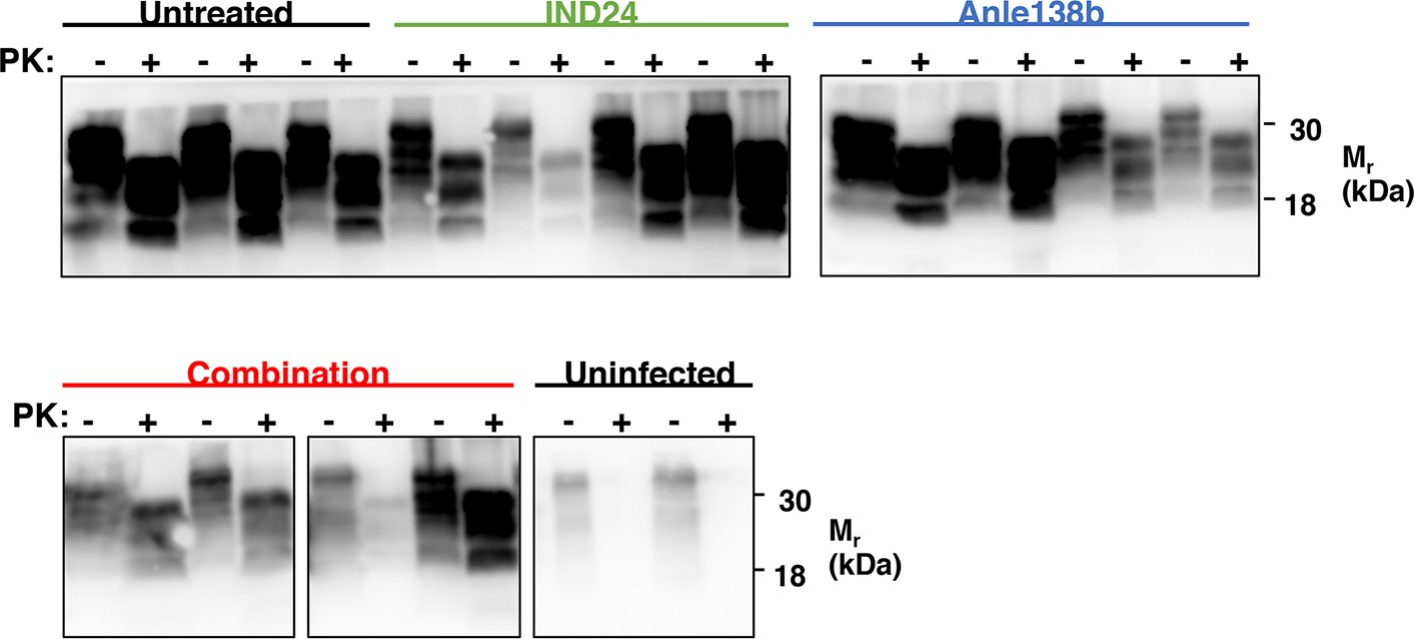
Proteinase K digestion of control- and experimentally-infected mouse brains. Western blots showing PrP^Sc^ in brain homogenates from RML-inoculated and uninfected mice from the indicated control or experimental condition harvested at terminal disease stage (or at 14 months of age for age-matched, uninfected control). Brain homogenate aliquots were treated with 64 μg/mL PK for 1 hr at 37°C, where indicated (+).

**Fig 3:**
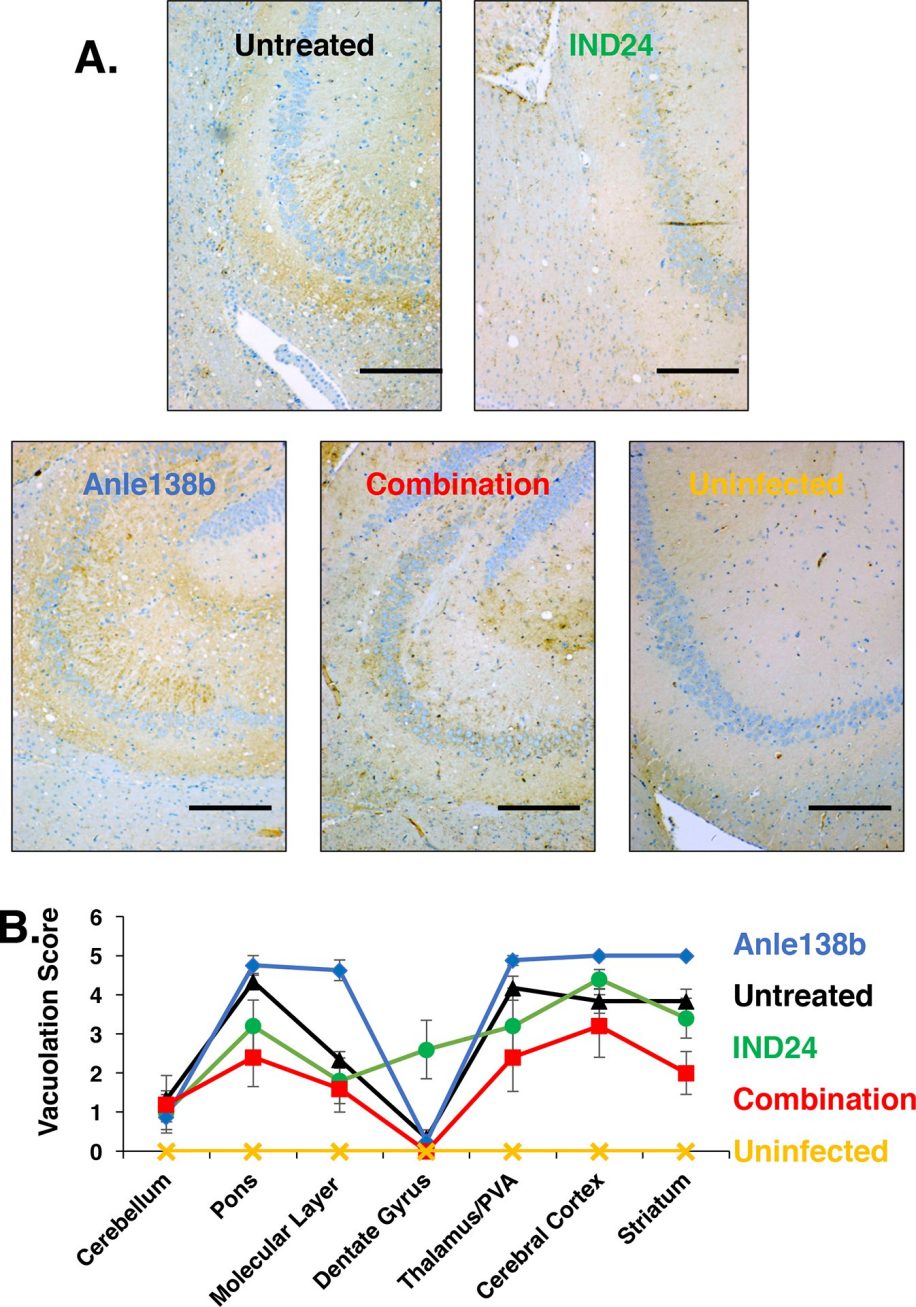
Neuropathology of mice treated with various drug regimens. **(A**) Representative microscopic images of brain sections of RML-inoculated and uninfected mice for each specified drug-treated or control group subjected to immunohistochemistry (IHC) with anti-PrP mAb 27/33 harvested at terminal disease stage (or at 14 months of age for age-matched, uninfected control). Scale bar = 200 μm. **(B)** Profiles of vacuolation scores of animals inoculated with a 10% RML brain homogenate and treated with no drug (black triangles, N = 6), IND24 (green circles, N = 5), Anle138b (blue diamonds, N = 8), a combination of IND24 and Anle138b (red squares, N = 5); or age-matched, uninfected animals (yellow crosses, N = 2). Mean values ± SEM are shown.

To test the hypothesis that combination therapy would be more effective than monotherapy in slowing the progression of prion infection, we treated RML-infected mice with three different oral drug regimens: one day post-inoculation (1) IND24 alone, (2) Anle138b, and (3) a combination of IND24 and Anle138b. As previously reported[8, 16], monotherapy with either IND24 or Anle138b significantly increased incubation times in RML-infected mice (~370 and ~320 days, respectively, *versus* ~150 days for untreated mice) **(Fig 1A, green circles and blue diamonds *vs*. black triangles; S1 Table)**. Surprisingly, combination therapy with both drugs did not produce a significant increase incubation time (~360 days) relative to monotherapy **(Fig 1A, red squares *vs*. green circles and blue diamonds; S1 Table)**. PrP^Sc^ accumulation in the brains of mice at terminal stage in all experimental groups was confirmed by PK digestion/Western blot **(Fig 2)** and immunohistochemistry (IHC) **(Fig 3A)**. These results indicate that the anti-prion effects of IND24 and Anle138b are neither synergistic nor additive.

### Combination therapy does not slow the rate of prion accumulation relative to monotherapy

**Fig 4:**
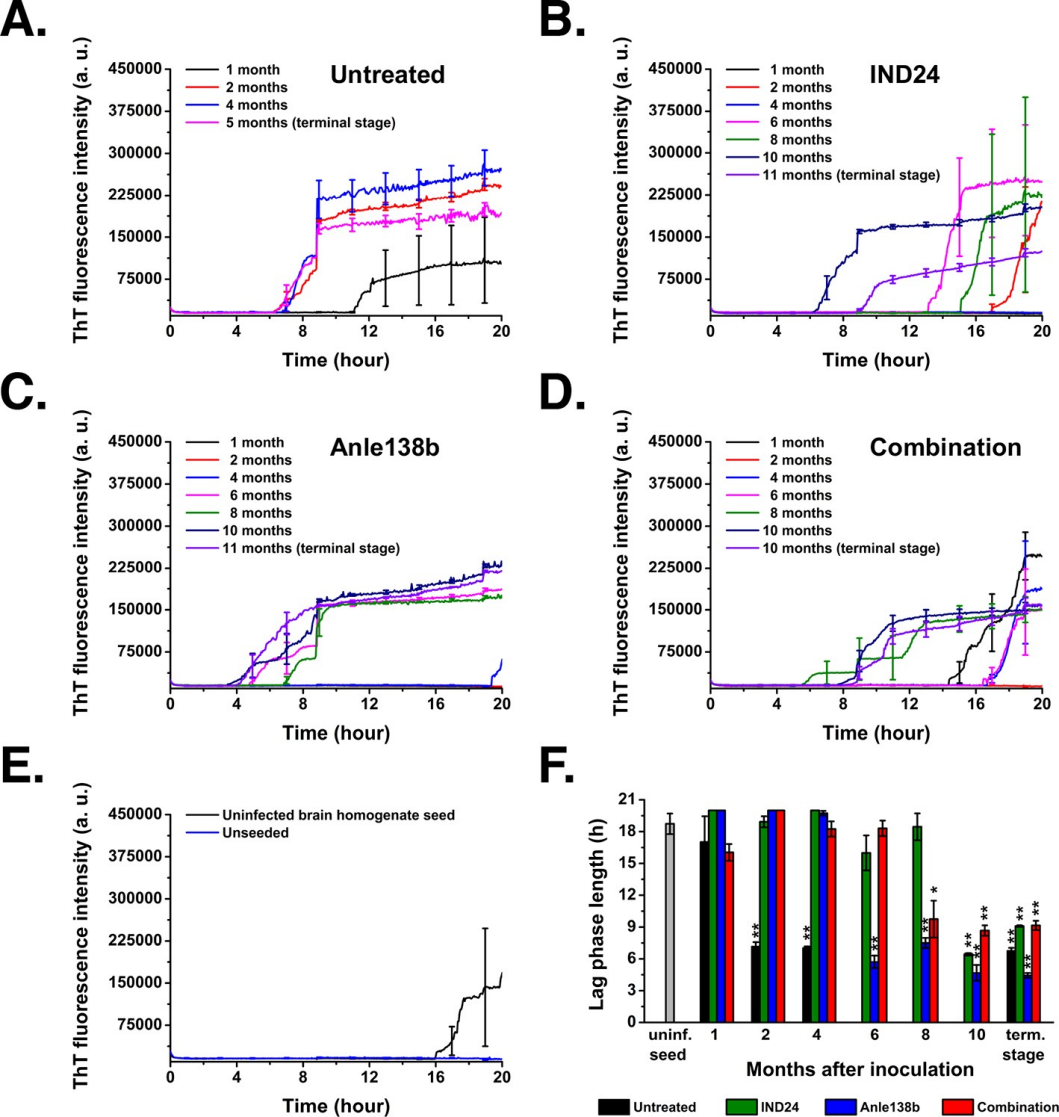
Analysis of prion formation over time in drug-treated mice brains by RT-QuIC. RT-QuIC reactions were seeded with a 10^−2^ dilution of 10% BH from mice inoculated with RML and treated with **(A)** no drug,**(B)** IND24, **(C)** Anle138b, **(D)** a combination of IND24 and Anle138b; or **(E)** 10^−2^ dilution of 10% BH from age-matched (14-month old), uninfected mouse. Data points are the average of technical triplicate samples. Mean ± S.E.M. are shown. Samples were tested simultaneously in the same sealed 96-well plate. (F) Lag phase lengths of samples seeded with RML-infected BHs were compared to control sample seeded with uninfected BH (grey column). Significantly shorter lag phases were observed in all RML-infected groups at different time points post-inoculation,(* p ≤ 0.05, ** p ≤ 0.01, n = 3). Terminal stage animals were harvest at the following times post-inoculation: Untreated = 5 months; IND24 and Anle138b = 11 months Combination = 10months.

We used a combination of IHC and real-time quaking-induced conversion (RT-QuIC) assays to evaluate the effect of combination therapy on the kinetics of PrP^Sc^ accumulation in the brains of infected mice. Mice in each drug treatment group were sacrificed at 2-month intervals, and their brains were harvested for both assays. Both assays showed that the rates of PrP^Sc^ accumulation were generally similar in mice treated with combination therapy and mice treated with single drugs, with very little RT-QuIC-seeding activity or positive IHC signal was detected during the first 8 months post-infection with any of the treatment regimens **(Fig 4B, 4C and 4D and S2 Fig, rows 2–4)**. In contrast, RT-QuIC seeding activity was detected 1-month post-infection and a positive IHC signal was detected 4 months post-infection in untreated controls **(Fig 4A and S2 Fig, top row)**. Similar results were obtained when PrP^Sc^ levels were analyzed by proteinase K digestion and western blot **(S3 Fig).** Thus, combination therapy does appear to reduce the rate of PrP^Sc^ formation but does not appear to be more effective than monotherapy in doing so.

### Emergence of prions resistant to combination therapy but not monotherapy

**Fig 5:**
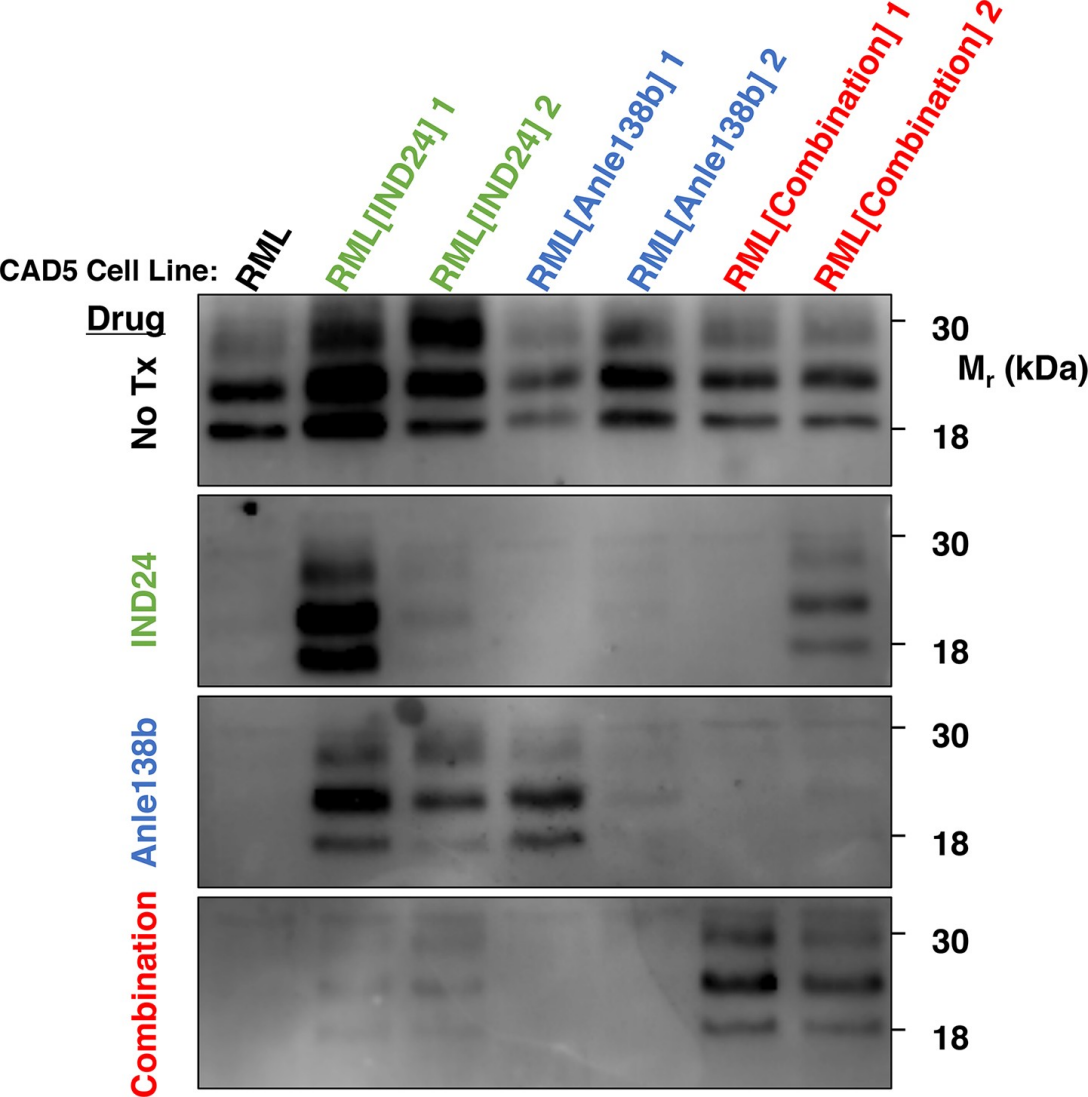
Formation of drug-resistant prions in CAD5 cells. Western blot showing the formation of PK-resistant PrP^Sc^ in the lysates from prion-infected CAD5 cells. Cell lines were treated with 10 μM of IND24, 10 μM of Anle138b, or a combination of 10 μM of IND24 and 10 μM of Anle138b. Each column represents an individual cell line, so there are two different cell lines (infected with brain homogenate from two different mice) for each drug treatment condition.

To further investigate the surprising lack of additional therapeutic benefit produced by the combination regimen, we performed cell-based drug susceptibility assays as previously described[8]. We first exposed cultured CAD5 cells to brain homogenates from individual mice in each treatment group (i.e. untreated, IND24 alone, Anle138b alone, and IND24/Anle138b combination), and confirmed successful infection of the cells by detection of PrP^Sc^ by PK digestion/Western blot **(S1 Fig)**. Cells from each group were then treated with each of the drug regimens to determine their *in vitro* resistance profiles. The results indicate that cells infected with combination-treated brain homogenates were resistant to combination therapy *in vitro* **(Fig 5, lanes 6–7, bottom panel)**. Remarkably, this was not associated with dual resistance to the individual drugs. One combination-treated isolate (RML[Combination]1) was susceptible to both IND24 and Anle138b **(Fig 5, lane 6, panels 3–4)**, while a second isolate (RML[Combination]2 taken from a different combination-treated animal) was susceptible to Anle138b **(Fig 5, lane 7, panel 4)**. Cells infected with monotherapy-treated brain homogenates exhibited different drug susceptibility profiles: IND24-treated isolates were resistant to IND24 and cross-resistant to Anle138b **(Fig 5, lanes 2–3)**, while Anle138b-treated isolates were only resistant to Anle138b **(Fig 5, lanes 4–5)**. Thus, combination therapy paradoxically prevents Anle138b resistance which would otherwise occur with either Anle138b or IND24 monotherapy.

To confirm the results of the *in vitro* drug susceptibility assays, we performed serial passage of prions from a mouse treated with the combination regimen into a new cohort of mice, which were then divided into different treatment groups. The incubation time data from this experiment validate that the prions from this mouse are indeed resistant to combination therapy and IND24, but susceptible to Anle138b **(Fig 1B, blue diamonds *vs*. red squares and green circles, and S2 Table)**. Interestingly, mice treated with either combination therapy or IND24 appeared to have slightly shorter incubation times than untreated mice **(Fig 1B, red squares and green circles *vs*. black triangles, and S2 Table)**, suggesting that these regimens may moderately promote the replication of combination-resistant prions. Taken together, our results show that combination-treated prions display a drug susceptibility profile that has not been previously reported, i.e. a pathogen that is resistant to combination therapy, but susceptible to monotherapy.

### Prions resistant to combination therapy display altered pattern of neurotropism

To determine whether the remarkable drug susceptibility profile of combination-resistant prions might be associated with a change in strain properties, we performed neuropathological analyses on the brains of mice from the first passage experiment (i.e. originally inoculated with stock RML prions). A comparison of vacuolation in different brain regions between the different treatment groups revealed that combination therapy induced the emergence of a profile that differed from those induced by IND monotherapy. Most notably, lower levels of vacuolation were observed in the dentate gyrus of mice treated with combination therapy compared to IND24 monotherapy **(Fig 3B, red squares *vs*. green circles)**. In addition, the combination regimen induced lower levels of vacuolation across all brain regions than Anle138b monotherapy **(Fig 3B, red squares *vs*. blue diamonds)**.

**Fig 6:**
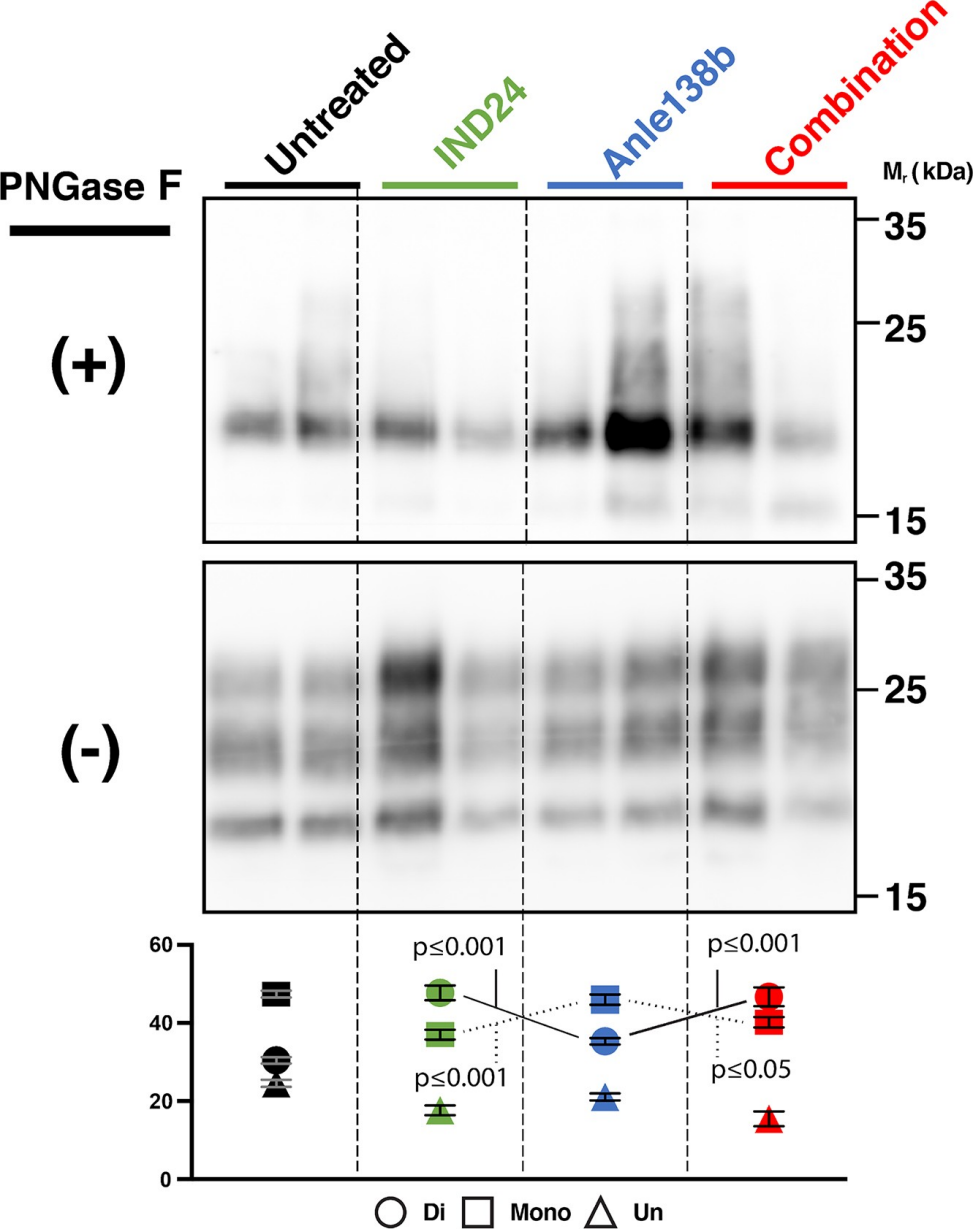
Analysis of glycoform distribution and electrophoretic mobility of PrP^Sc^ molecules in the brains of chemotherapy treated mice. Western blots of brain homogenates prepared from animals inoculated with RML and treated with (top) or without (bottom) PNGase F. Proportion of glycoforms quantified from the western blot without PNGase is below in which diglycosylated PrP^Sc^ = circles; monoglycosylated PrP^Sc^ = squares; or unglycosylated PrP^Sc^ = triangles. Samples were all subjected to limited proteolysis. Samples were deglycosylated by treatment with PNGase F, indicated as (+), before SDS/PAGE. Quantification of each glycoform species was performed from western blots using the image taken preceding any signal saturation. Data points shown are the mean of biological duplicates with technical quintuplets. Mean ± S.E.M. are shown for each point. P-values represented are from individual t-tests were calculated between Anle138b and (either IND24 or combination regimen) treated animals for mono and di-glycosylated species.

**Fig 7:**
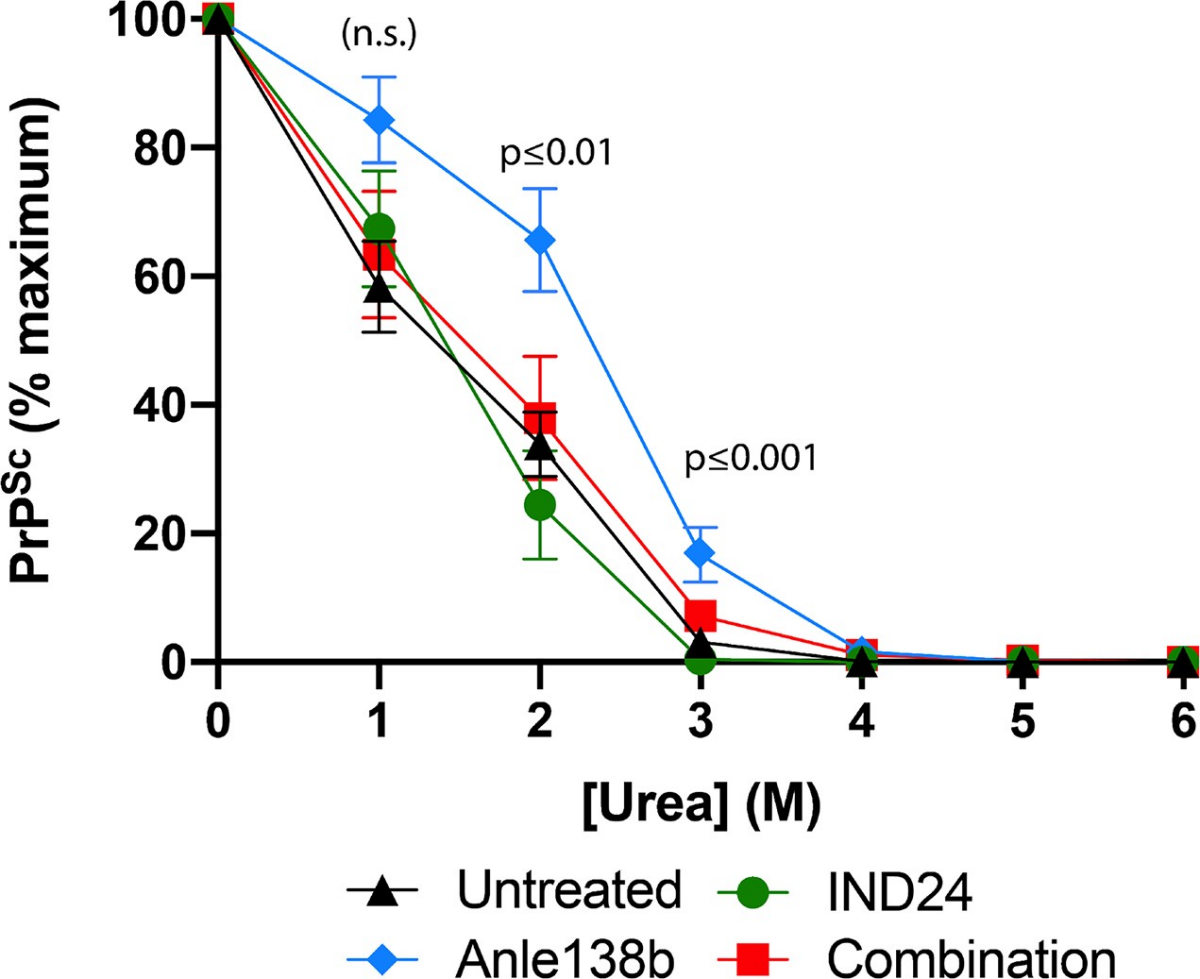
Analysis of PrP^Sc^ conformational stability. Urea denaturation assay showing PrP^Sc^ levels in samples of brain homogenates prepared from animals inoculated with RML. Animals were treated with no drug, black triangles; IND24, green circles; Anle138b, blue diamonds; or a combination of both IND24 and Anle138b, red squares. Western blot images were quantified using the image taken preceding any signal saturation and data was normalized relative to the 0M Urea lane. Data points are the average of biological duplicates each with technical quadruplicate samples. Mean ± S.E.M. are shown for each point. Significant differences conformational stability at various timepoints among treatments was calculated using one-way ANOVA and multiple-testing corrected using Tukey’s range test.

We also performed biochemical assays on prions from each experimental group. We did not observe any obvious differences in PrP^Sc^ electrophoretic mobility between the experimental groups **(Fig 6, top panel)**. However, Anle138b treated mice appeared to have a higher ratio of mono:di -glycosylated PrP^Sc^ than mice treated with IND24 or the combination regimen both of which had di-glycosylated PrP^Sc^ as the predominant species instead of mono-glycosylated as with Anle138b treated mice **(Fig 6, lower panel)**. Likewise, urea denaturation curves showed that PrP^Sc^ conformation is more stable in Anle138b-treated mice than in mice treated with either IND24 or combination therapy **(Fig 7, blue diamonds *vs*. red squares and green circles)**.

## Discussion

The major finding of this work is that infectious prions can develop resistance to a combination drug regimen despite maintaining susceptibility to an individual component of the regimen. To our knowledge, this phenomenon has not been previously reported. Conventional infectious pathogens and cancer cells that are resistant to combination chemotherapy are invariably resistant to all of the individual drugs in the regimen, and multi-drug resistance in these situations is due the accumulation of individual drug resistance mutations.

### Resistance to combination chemotherapy but not Anle138b monotherapy

We observed that combination therapy with Anle138b and IND24 induces the emergence of prions that are resistant to the combination regimen, but susceptible to Anle138b monotherapy. Maintenance of Anle138b susceptibility is very surprising because both Anle138b and IND24 induce Anle138b resistance when administered individually. Specifically, our *in vitro* drug susceptibility assays indicate that Anle138b monotherapy induces its own resistance while IND24 monotherapy induces cross-resistance to Anle138b. Thus, when administered concurrently, each drug appears to inhibit the ability of the other drug to induce Anle138b resistance. Moreover, it is difficult to envision how Anle138b susceptibility can be maintained even though the resulting prions develop resistance to the combination regimen (which includes Anle138b).

### Potential mechanism of selective resistance to combination therapy

Unlike conventional pathogens and cancer cells, which contain nucleic acid genomes and develop drug resistance by acquiring specific mutations, infectious prions appear to develop single drug resistance through the selection of alternative PrP^Sc^ conformers from a cloud of PrP^Sc^ quasi-species. We examined whether a similar mechanism might also be responsible for combination drug resistance by analyzing the neuropathological profiles and PrP^Sc^ conformation in each of the treatment groups of the initial and serial passage experiments. These analyses showed that the prions induced by combination therapy have different pathological and biochemical properties from those induced by monotherapy, suggesting that resistance to combination therapy could also be caused by selection of a specific prion strain associated with an alternative PrP^Sc^ conformation.

Based on our results, if resistance to combination therapy is indeed caused by selection of a novel prion strain, the resulting PrP^Sc^ conformation must simultaneously satisfy the following constraints: (1) it should be inhibited by Anle138b since monotherapy with this drug remains effective; (2) this inhibitory effect of Anle138b should be antagonized by IND24 since combination therapy is ineffective; and (3) it should not be directly inhibited by IND24 since IND24 therapy is ineffective.

It is surprising that the strain induced by combination therapy differs from the strain induced by IND24 monotherapy, since both of these strains appear to be resistant to the combination regimen. However, the two strains can be distinguished on the basis of (1) resistance to Anle138b monotherapy and (2) vacuolation profiles. Apparently, the presence of Anle138b in the combination regimen suppresses emergence of the IND24-induced cross-resistant strain, whereas absence of Anle138b promotes its emergence. One possible explanation for this phenomenon is that Anle138b helps IND24 to positively catalyze the replication of the combination therapy-induced strain; it has been previously shown that a single compound can inhibit one prion strain and promote a different strain[11]. This potential explanation is supported by the serial passage incubation data, which show that re-exposure to combination therapy actually shortens incubation time in treated mice compared to untreated animals. However, this explanation is weakened by the observation that IND24 monotherapy also promotes a faster incubation time in serial passage mice. An alternative explanation is that IND24 and Anle138b may work by inducing alternative PrP^Sc^ conformers during the replication process itself (perhaps by binding to PrP^Sc^ to cause “deformed templating”[17]) rather than by selection of pre-existing conformers.

It is important to note that the difference in drug resistance patterns and neuropathological profiles between prions induced by IND24 monotherapy and combination therapy also exclude the possibility that the IND24 and Anle138b might simply be competing for the same binding site on PrP. The difference in strain properties between prions induced by monotherapy with IND24 *versus* Anle138b also argue against this trivial explanation.

It is also worth noting that Anle138b contains a methylenedioxyphenyl group, which can inhibit cytochrome P450. Therefore, the level of IND24 exposure in the combination-treated mice may be higher than the level of exposure in mice treated with IND24 monotherapy.

We observed some variation in the total amount of protease-resistant PrP^Sc^ **(Fig 2)** and the pattern of drug-resistance **(Fig 5)** between individual mice treated with the same regimen. Similar variability in the accumulation of protease-resistant PrP^Sc^ was previously observed in terminally ill mice treated either with IND24 or IND81[8]. We do not know the reason for this naturally occurring heterogeneity, but it is possible that multiple PrP^Sc^ conformers are resistant to each compound. Different PrP^Sc^ conformers, each with their own degree of protease-resistance drug-resistance profile, might be selected upon treatment with the same drug in different mice.

### Implications for future therapeutic strategies

Our results indicate that prions may be even more malleable than appreciated. Combination chemotherapy is typically an effective strategy against conventional pathogens and cancer cells because: (1) the probability of harboring multiple drug resistance mutations is much lower than the probability of harboring any single mutation, and (2) the need to harbor multiple drug resistance mutations can reduce replication fitness. In contrast, we observed that infectious prions can develop resistance to a combination regimen without necessarily having resistance to the individual components. Moreover, the resulting combination regimen-resistant strain appeared to replicate just as quickly as treatment-naïve prions. Taken together, these observations suggest that prions may be able to evade combination therapy more easily than conventional pathogens or cancer cells. Another recent report showed that combining autophagy stimulators and cellulose esters did not provide any additional survival benefit over monotherapy in prion-infected mice[18]. However, the mechanism by which prions circumvented the combination regimens used in that study may differ from the strain adaptation in response to IND24 and Anle138b observed in this study. In conclusion, it may be difficult to use conventional combination therapy to combat prions due to their malleability, manifested here by the formation of a pathogen that is uniquely resistant to a combination therapy but not monotherapy.

## Materials and methods

### Ethics statement

The Guide for the Care and Use of Laboratory Animals of the National Research Council was strictly followed for all animal experiments. All experiments mice in this study were conducted in accordance with protocol supa.su.1 as reviewed and approved by Dartmouth College’s Institutional Animal Care and Use Committee, operating under the regulations/guidelines of the NIH Office of Laboratory Animal Welfare (assurance number A3259-01).

### Animal inoculations, drug treatment, diagnosis, and neuropathology

Intracerebral inoculation and diagnosis of prion disease were performed as described[19] with the following modifications: Brain homogenate samples (10% (w/v) in PBS) were spun for 30 sec at 200 *x g* to remove nuclear debris, and the supernatant was collected and used as the inoculum. The inoculum volume used was 30 μL. RML was a gift from the Prusiner Lab (UCSF) and passaged in CD-1 mice prior to use as the initial inoculum. Female CD-1 mice were obtained from Charles River Laboratories (Wilmington, MA, USA) and inoculated between 4–5 weeks of age. Starting one day following inoculation, animals were fed Teklad chow (Envigo, Madison, WI) formulated by Envigo to contain either 280 mg compound/kg body weight/day IND24, 400 mg/kg/day Anle138b, or 280 mg/kg/day IND24 plus 400 mg/kg/day Anle138b. IND24 and Anle138b were synthesized by Chemveda Life Sciences (San Diego, CA, USA). Prior to inoculation, animals were fed Teklad chow alone. Neuropathology was performed as previously described[20], using primary mAb 27/33 at a 1:1000 dilution and a Biocare Mouse on Mouse Horseradish Peroxidase Polymer (Biocare Medical, Pacheco, CA) for the immunohistochemical detection of PrP.

### Proteinase K digestion and detection of PrP^Sc^ in experimentally infected brains

Formation of PrP^Sc^ was monitored by digestion of BHs [10% (w/v) in PBS] with PK followed by western blotting. Samples were digested in a reaction containing 64 μg/mL PK (unless otherwise specified), 2% (v/v) Tween-20 (Fisher Scientific, Hampton, NH), 2% (v/v) NP-40 (Fisher Scientific, Hampton, NH), and 2% (w/v) n-Octyl-β-D-Glucopyranoside (Anatrace, Maumee, OH) at 37°C with shaking at 750 r.p.m. for 1 hr. Digestions were quenched by adding SDS-PAGE loading buffer and heating to 95°C for 15 min. SDS-PAGE and western blotting were performed as described previously [19] using mAb 27/33. Twenty microliters of a brain homogenate were subjected to PK digestion. The minus (-) PK lane is used to determine the fraction of PrP that has been converted to PrP^Sc^ in the brain. The minus PK lane contains the same volume (20 μL) of BH as a PK-digested sample.

### CAD5 cell culture

CAD5 cells were a gift from the Prusiner Lab (University of California, San Francisco). CAD5 cells were maintained at 37°C in Opti-MEM I Reduced Serum Medium (Fisher Scientific, Hampton, NH, USA) supplemented with 9% HyClone Bovine Growth Serum (VWR, Radnor, PA, USA) and 100 units Penicillin-Streptomycin Solution, 100x (Corning Inc., Corning, NY, USA). Dividing cells were plated at 10% confluency and were split 1:10 when confluent.

### Prion Infection of CAD5 cells

CAD5 cells were grown to confluency and split 1: 10 into 6-well plates. Two-hundred microliters of a 10% brain homogenate solution ((w/v) in PBS) was added to 1.8 mL of media. Cells were grown for 4 days to confluency then split 1:10 into fresh media. Once cells reached confluency, each well was harvested in 1.2 mL lysis buffer (20 mM Tris pH 8.0, 100 mM NaCl, 0.5% Nonidet P-40, and 0.5% sodium deoxycholate). Tubes were spun for 5 sec at 500 *x g* to pellet DNA, and 1 mL of supernatant was removed for processing. Formation of PrP^Sc^ was monitored by digestion of lysates with PK followed by western blotting. Samples were digested in a reaction containing 20 μg/mL PK for 1 hr at 37°C with shaking at 300 r.p.m. The digest was quenched by addition of PMSF to 2mM. Tubes were spun at 100,000 *x g* for 1 hr at 4 C and the supernatant was removed. Pellets were resuspended in SDS sample buffer and boiled for 15 min at 95°C. SDS-PAGE and western blotting were performed as described previously[19] using mAb 27/33. The minus (-) PK lane is used to determine the amount of PrP^C^ in the cells relative to the amount that was converted to PrP^Sc^. The minus PK lane contains 40 μL of un-spun lysate, one-tenth the volume of un-spun lysate in the plus (+) PK lane.

### Drug treatment of CAD5 cells

Prion-infected CAD5 cells were grown to confluency and split 1:10 into 3 mL plates. Cells were treated for 5 days with media containing drug (either 10 μM IND24, 10 μM Anle138b, or 10 μM IND24 and 10 μM Anle138b). Media was exchanged on the third day and replaced with media containing drug. Cells were harvested on the fifth day in 1.0 mL lysis buffer (20 mM Tris pH 8.0, 100 mM NaCl, 0.5% Nonidet P-40, and 0.5% sodium deoxycholate). Tubes were spun for 5 sec at 500 *x g* to pellet DNA, and 0.9 mL of supernatant was removed for processing. Formation of PrP^Sc^ was monitored by digestion of lysates with PK followed by western blotting. Samples were digested in a reaction containing 20 μg/mL PK for 30 min at 37°C with shaking at 350 r.p.m. The digest was quenched by addition of PMSF to 2 mM. A phosphotungstic acid (PTA) precipitation of the digested lysates was performed to concentrate PrP^Sc^. Seventy-five microliters of 10% PTA pH 7.0 and 33 μL of 30% Sarkosyl were added to the samples for a final concentration of 0.75% PTA and 1% Sarkosyl. Samples were incubated for 3 hr at 37°C with shaking at 350 r.p.m. then centrifuged at 4°C at 18,000 *x g* for 1 hr. The supernatants were discarded, and pellets were resuspended in SDS sample buffer and boiled for 15 min at 95°C. SDS-PAGE and western blotting were performed as described previously[19] using mAb 27/33.

### Real-Time Quaking Induced Conversion (RT-QuIC) Assay

RT-QuIC reactions were carried out as described previously[21], with the following modifications. Lyophilized mouse recPrP was resuspended in 10 mM sodium phosphate (pH 5.8) to a concentration of 0.5 mg/mL. The resuspended protein was filtered through a 0.22-μm syringe-driven filter, and the concentration was adjusted using 10 mM sodium phosphate (pH 5.8) to a concentration of 0.3 mg/mL. The resuspended protein was then diluted in a reaction buffer (10 mM sodium phosphate buffer pH 7.4, 130 mM NaCl, 10 μM ThT, 1 mM EDTA, and 0.001% SDS) to a final concentration of 0.1 mg/mL. Ninety-eight microliters of this reaction mixture were added to each well of a black-walled 96-well plate with a clear bottom with 2 μL of seed. Ten-fold serial dilutions of seeds were created in PBS and 0.025% (v/v) SDS. The plate was sealed and incubated at 42°C with 90-sec intervals of orbital shaking at 920 r.p.m. followed by 90 sec of rest in a FilterMax F5 Multi-Mode Microplate Reader (Molecular Devices, San Jose, CA). ThT fluorescence measurements (430 +/- 35-nm excitation and 485 +/- 20-nm emission) were taken every three min. Experimental samples were run in technical triplicate. Data analysis was performed using OriginPro 8.5.0 (OriginLab Corporation, Northampton, MA). Data were expressed as baseline-subtracted means ± standard error of the mean (SEM).

### Analysis of RT-QuIC lag phase lengths

Amyloid fibril formation often follows sigmoidal growth kinetics, with consecutive steps of nucleation, elongation, and saturation [22]. Under identical experimental conditions, using equal volumes and concentrations of monomeric prion protein, the lag time of PrP aggregation depends on scrapie dose and morphological distribution of aggregates in seeding material [23, 24].

Lag time values in RT-QuIC experiments were obtained by linear fitting to the baseline of the lag phase and to the steepest region of growth phase curve. Lag phase length was defined by the intersection of the two lines [22, 25]. In the case of samples where the lag time could not be obtained (since it exceeded the duration of the experiment), lag phase length was considered to be 20 hrs long. Obtained data was analyzed by one-way ANOVA and Tukey’s range test [26].

### Urea denaturation assay

Fifteen microliters of 10% (wt/vol) brain homogenate was mixed with 60 μL of various urea/0.25% (vol/vol) Triton X-100 solution containing 25mM Tris, pH 7.5 to obtain final urea concentrations between 0 and 6 M. Samples then were incubated at 70°C for 3 h with shaking at 750 r.p.m. Next, 50 μL of 50 mM Mops (pH 7.0) containing 330 mM NaCl, 1% (vol/vol) Triton X-100, 5 mM CaCl2 and 125 μg/mL proteinase K was added, samples were incubated at 37°C for 60 min, with shaking at 750 r.p.m. PK reactions were quenched with the addition of 5mM PMSF. Then 100 μL of 4X SDS sample buffer was added, and samples were boiled for 10 min at 95°C. SDS-PAGE and western blotting were performed as described previously [19] using mAb 27/33. Western blots were quantified using Image Studio Lite (LI-COR Biosciences).

### Enzymatic deglycosylation

Seventy-five microliters of 10% (wt/vol) brain homogenate was added to 25 μL of PBS, 2% (vol/vol) Triton X-100 containing 40 μg/mL proteinase K, and samples were shaken at 750 r.p.m. for 30 min at 37°C. After incubation, 10 μL of 100 mM PMSF (in 100% EtOH) was added, and the sample was vortexed and incubated at room temperature for 10 min. Next, samples were diluted with 895 μL PBS, 0.5% (vol/vol) Triton X-100 and were centrifuged at 100,000 × g for 60 min at 4°C, and supernatants were discarded. Pellets then were resuspended in 10 μL of 10X glycoprotein denaturation buffer, subjected to three 30-s bursts of sonication, and boiled at 95°C for 10 min. Samples then were diluted with 80 μL water, and sonication and boiling were repeated. Next, samples were cooled to room temperature, and 13 μL each of 10X G7 reaction buffer and 10% (vol/vol) Nonidet P-40 and 5 μL of peptide:N-glycosidase F (PNGase F) were added to each sample. Samples were shaken at 250 r.p.m. overnight at 37°C. Reactions were stopped by the addition of 50 μL of 4X SDS sample buffer and boiling at 95°C for 10 min. SDS-PAGE and western blotting were performed as described previously [19] using mAb 27/33. Western blots were quantified using Image Studio Lite (LI-COR Biosciences) and represented as glycoform ratios. Differences in glycoform ratios were determined by performing one-way ANOVA test for each glycoform species and post-hoc analyzed with Tukey’s range test to correct for multiple testing.

## Supporting information

S1 Fig Generation of prion-infected CAD5 cell lines. Western blot showing the formation of PK-resistant PrP^Sc^ in lysates from uninfected CAD5 cells and cells infected with the brain homogenate from terminally-ill mice from the indicated drug treatment group. Each sample represents an individual cell line, so there are three different cell lines (infected with brain homogenate from three different mice) for each experimental condition Samples were treated with 20 μg/mL PK for 1 hr at 37°C, where indicated (+).
(TIF)

S2 Fig Histopathology of brains harvested at various time points post-inoculation. Representative microscopic images of brain sections of RML-inoculated mice subjected to immunohistochemistry (IHC) with anti-PrP mAb 27/33 harvested at the indicated time point post-inoculation for each specified drug-treated or untreated control group. Scale bar = 200 μm.
(TIF)

S3 Fig Proteinase K digestion of control- and experimentally-infected mouse brains. Western blots showing PrP^Sc^ in brain homogenates from various groups of RML-inoculated or uninfected mice harvested at specific time points, as indicated. Brain homogenate aliquots were treated with 64 μg/mL PK for 1 hr at 37°C, except where indicated (-PK). The brain of the age-matched, uninfected mouse was harvested at 14 months of age. Asterisks (*) indicate endpoint mice that showed significant clinical signs of infection.
(TIF)

S1 Table RML inoculations of drug-treated mice. Mice were inoculated with a 10–1 dilution of 10% RML brain homogenate (or a diluent control) and fed diets containing regular chow (Untreated), IND24, Anle138b, or a combination of IND24 and Anle138b. IP = incubation period until appearance of clinical symptoms. SEM = Standard error of the mean. n/n0 = number of animals with clinical symptoms/ total number of animals in the group.
(DOCX)

S2 Table Combination-treated BH inoculations of drug-treated mice. Mice were inoculated with a 10–1 dilution of 10% brain homogenate from a mouse inoculated originally with RML and treated with a combination of IND24 and Anle138b. Mice were fed diets containing regular chow (Untreated), IND24, Anle138b, or a combination of IND24 and Anle138b. IP = incubation period until appearance of clinical symptoms. SEM = Standard error of the mean. n/n0 = number of animals with clinical symptoms/ total number of animals in the group.
(DOCX)
