# Correction: Emergence of Prions Selectively Resistant to Combination Drug Therapy

**DOI:** 10.1371/journal.ppat.1011919

**Published:** 2024-01-08

**Authors:** Cassandra M. Burke, Kenneth M. K. Mark, Judit Kun, Kathryn S. Beauchemin, Surachai Supattapone

An additional affiliation is missing for the third author. Judit Kun is also affiliated with: Department of Biochemistry, Eötvös Lorna University, Budapest, Hungary.
