# Supplementary figures and images for: Emergence of prions selectively resistant to combination drug therapy
Source: PLoS Pathog. 2020 May 18;16(5):e1008581. doi: 10.1371/journal.ppat.1008581 (PMC7259791; doi:10.1371/journal.ppat.1008581)

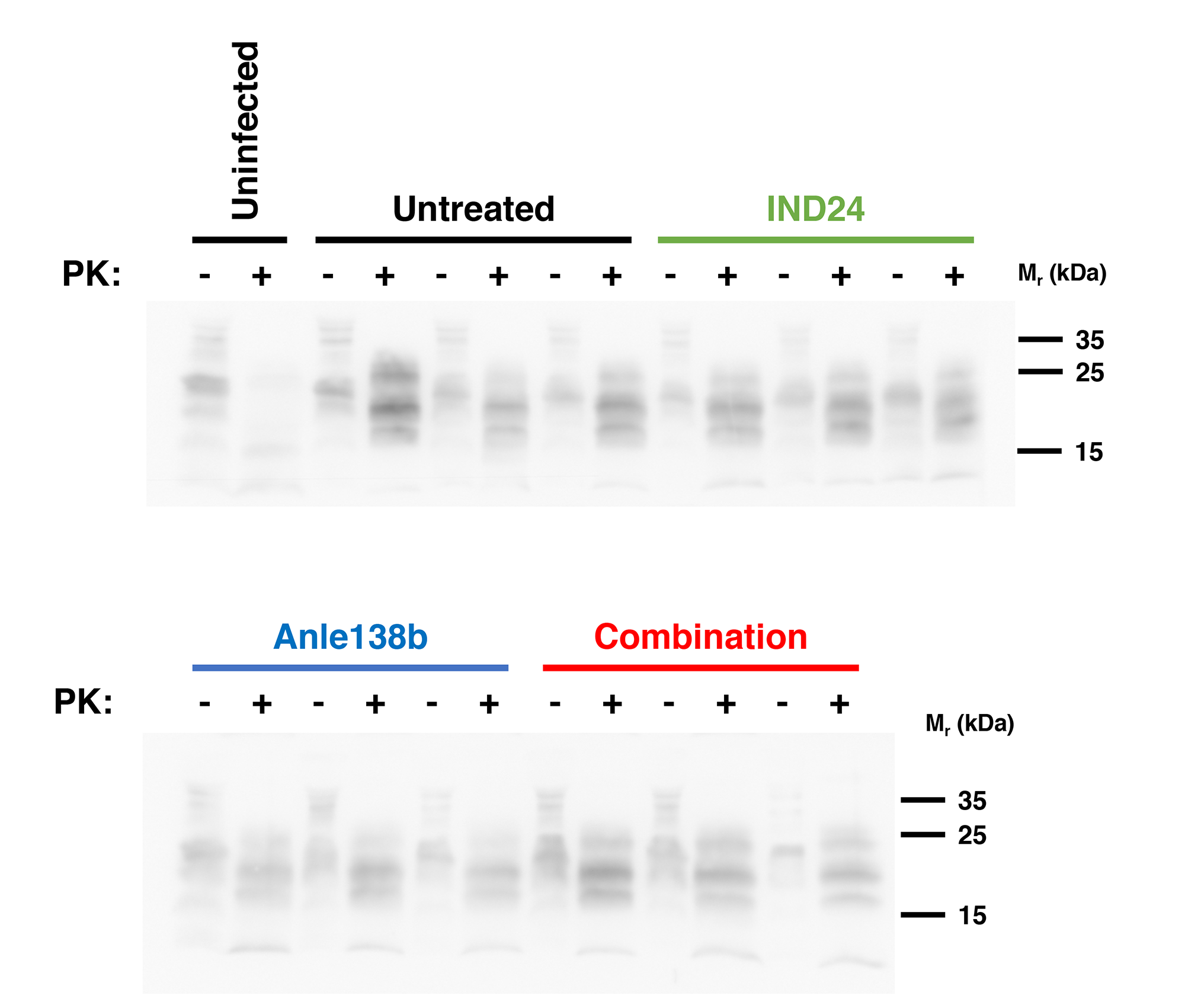

Supplement: S1 Fig — Western blot showing the formation of PK-resistant PrPSc in lysates from uninfected CAD5 cells and cells infected with the brain homogenate from terminally-ill mice from the indicated drug treatment group. Each sample represents an individual cell line, so there are three different cell lines (infected with brain homogenate from three different mice) for each experimental condition Samples were treated with 20 μg/mL PK for 1 hr at 37°C, where indicated (+). (TIF) [file ppat.1008581.s001.tif]

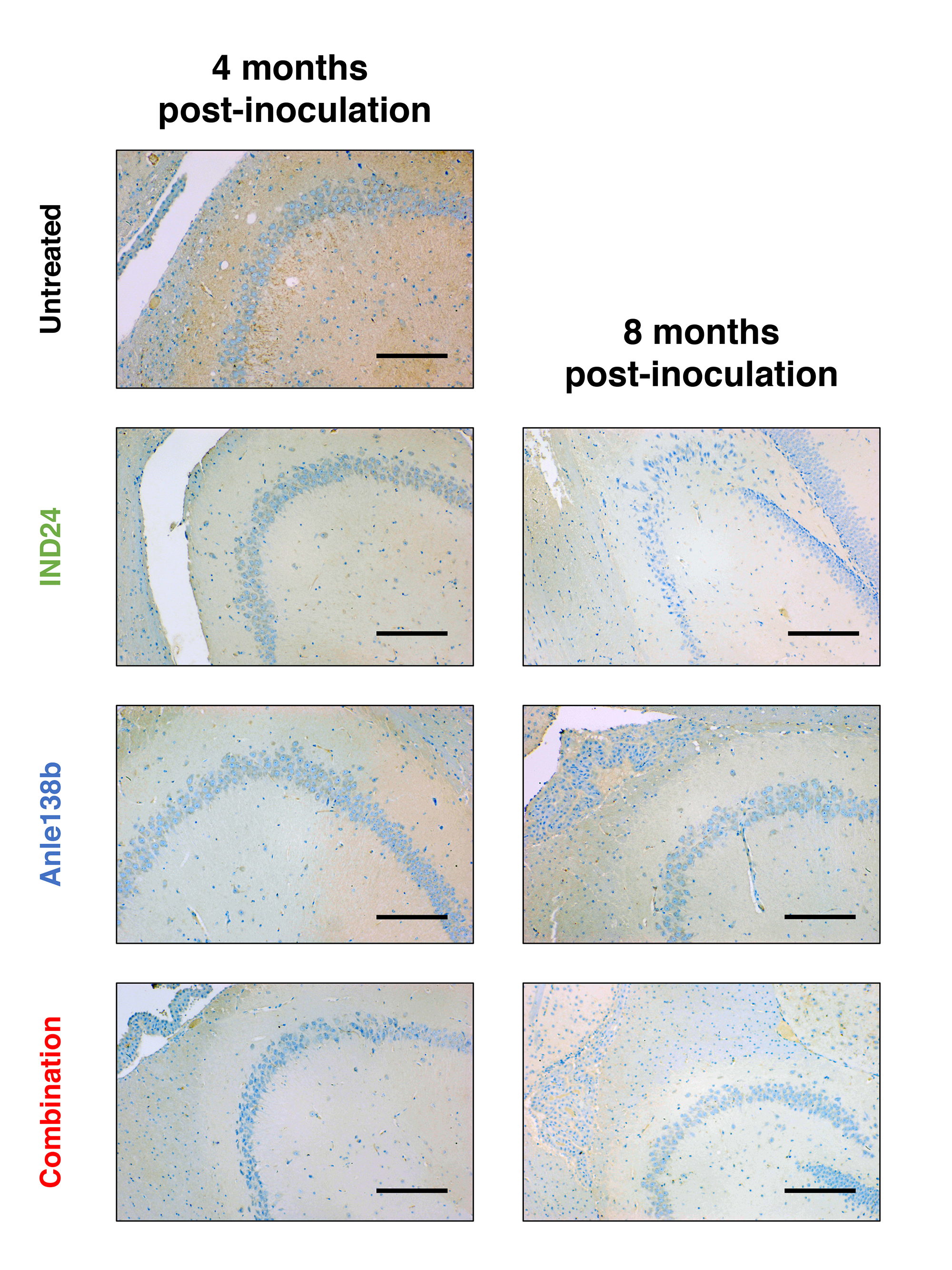

Supplement: S2 Fig — Representative microscopic images of brain sections of RML-inoculated mice subjected to immunohistochemistry (IHC) with anti-PrP mAb 27/33 harvested at the indicated time point post-inoculation for each specified drug-treated or untreated control group. Scale bar = 200 μm. (TIF) [file ppat.1008581.s002.tif]

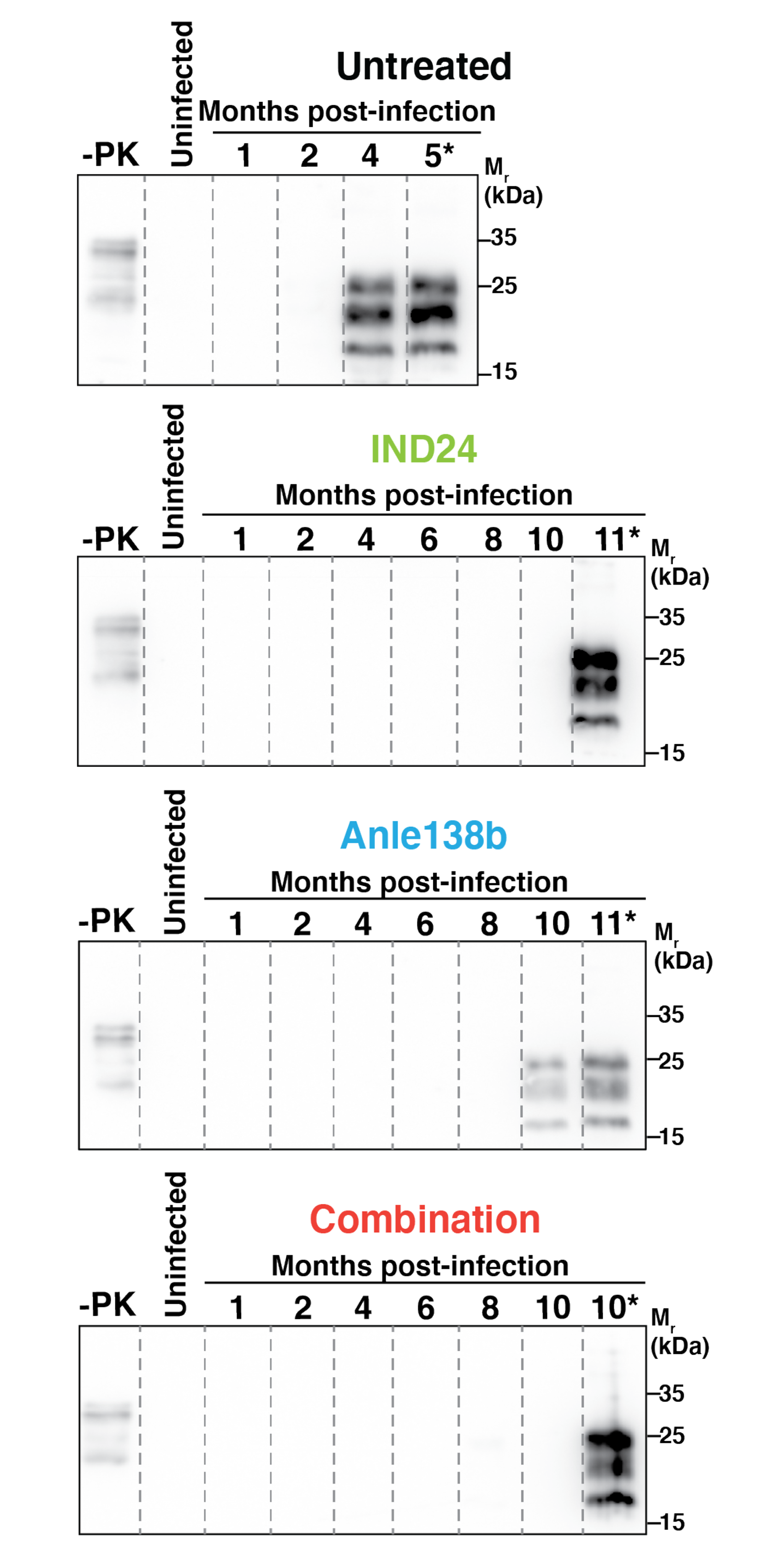

Supplement: S3 Fig — Western blots showing PrPSc in brain homogenates from various groups of RML-inoculated or uninfected mice harvested at specific time points, as indicated. Brain homogenate aliquots were treated with 64 μg/mL PK for 1 hr at 37°C, except where indicated (-PK). The brain of the age-matched, uninfected mouse was harvested at 14 months of age. Asterisks (*) indicate endpoint mice that showed significant clinical signs of infection. (TIF) [file ppat.1008581.s003.tif]
