# Supplementary material for: Emergence of prions selectively resistant to combination drug therapy
Source: PLoS Pathog. 2020 May 18;16(5):e1008581. doi: 10.1371/journal.ppat.1008581 (PMC7259791; doi:10.1371/journal.ppat.1008581)
Supplement: S1 Table — Mice were inoculated with a 10–1 dilution of 10% RML brain homogenate (or a diluent control) and fed diets containing regular chow (Untreated), IND24, Anle138b, or a combination of IND24 and Anle138b. IP = incubation period until appearance of clinical symptoms. SEM = Standard error of the mean. n/n0 = number of animals with clinical symptoms/ total number of animals in the group. (DOCX) [file ppat.1008581.s004.docx]

**Supplemental Table S1: RML inoculations of drug-treated mice**

Mice were inoculated with a 10^-1^ dilution of 10% RML brain homogenate and fed diets containing regular chow (Untreated), IND24, Anle138b, or a combination of IND24 and Anle138b. IP = incubation period until appearance of clinical symptoms. SEM = Standard error of the mean. n/n_0_ = number of animals with clinical symptoms/ total number of animals in the group.

| **Experiment Group** | **n/n_0_** | **Mean IP (days)** | **± SEM** |
| --- | --- | --- | --- |
| Diluent Control | 0/2 | >450 |  |
| Untreated | 9/9 | 150 | ±1.7 |
| IND24 | 8/8 | 377 | ± 14.9 |
| Anle138b | 9/9 | 318 | ±12.4 |
| Combination | 11/11 | 359 | ±18.6 |
|  |  |  |  |
