# Supplementary material for: Emergence of prions selectively resistant to combination drug therapy
Source: PLoS Pathog. 2020 May 18;16(5):e1008581. doi: 10.1371/journal.ppat.1008581 (PMC7259791; doi:10.1371/journal.ppat.1008581)
Supplement: S2 Table — Mice were inoculated with a 10–1 dilution of 10% brain homogenate from a mouse inoculated originally with RML and treated with a combination of IND24 and Anle138b. Mice were fed diets containing regular chow (Untreated), IND24, Anle138b, or a combination of IND24 and Anle138b. IP = incubation period until appearance of clinical symptoms. SEM = Standard error of the mean. n/n0 = number of animals with clinical symptoms/ total number of animals in the group. (DOCX) [file ppat.1008581.s005.docx]

**Supplemental Table S2: Combination-treated BH inoculations of drug-treated mice**

Mice were inoculated with a 10^-1^ dilution of 10% brain homogenate from a mouse inoculated originally with RML and treated with a combination of IND24 and Anle138b. Mice were fed diets containing regular chow (Untreated), IND24, Anle138b, or a combination of IND24 and Anle138b. IP = incubation period until appearance of clinical symptoms. SEM = Standard error of the mean. n/n_0_ = number of animals with clinical symptoms/ total number of animals in the group.

| **Experiment Group** | **n/n_0_** | **Mean IP (days)** | **± SEM** |
| --- | --- | --- | --- |
| Untreated | 7/7 | 214 | ± 6 |
| IND24 | 5/5 | 181 | ± 4 |
| Anle138b | 6/6 | 273 | ± 8 |
| Combination | 8/8 | 191 | ± 4 |
|  |  |  |  |
